# Virological evaluation of natural and modified attapulgite against porcine epidemic diarrhoea virus

**DOI:** 10.1186/s12985-024-02396-w

**Published:** 2024-05-30

**Authors:** Tianmin Wang, Yuan Wen, Bingxu Qian, Fang Tang, Xiaorong Zhang, Xiulong Xu, Yanmin Zhou, Jianjun Dai, Aiqin Wang, Feng Xue

**Affiliations:** 1 MOE Joint International Research Laboratory of Animal Health and Food Safety, College of Veterinary Medicine, Nanjing Agricultural University, Nanjing, 210095 China; 2 Jiangsu Co-Innovation Center for the Prevention and Control of Animal Infectious Disease and Zoonoses, College of Veterinary Medicine, Yangzhou University, Yangzhou, 225104 China; 3 College of Animal Science and Technology, Nanjing Agricultural University, Nanjing, 210095 China; 4 China Pharmaceutical University, Nanjing, 211198 China; 5 Key Laboratory of Clay Mineral Applied Research of Gansu Province, Center of Eco-material and Green Chemistry, Lanzhou Institute of Chemical Physics, Chinese Academy of Sciences, Lanzhou, 730099 China

**Keywords:** Attapulgite, Porcine epidemic diarrhea virus, Antiviral material, Transmission electron microscopy of viruses

## Abstract

**Background:**

The Porcine Epidemic Diarrhea Virus (PEDV) has caused significant economic losses in the global swine industry. As a potential drug for treating diarrhea, the antiviral properties of attapulgite deserve further study.

**Methods:**

In this study, various methods such as RT-qPCR, Western blot, viral titer assay, Cytopathic Effect, immunofluorescence analysis and transmission electron microscopy were used to detect the antiviral activity of attapulgite and to assess its inhibitory effect on PEDV.

**Results:**

When exposed to the same amount of virus, there was a significant decrease in the expression of the S protein, resulting in a viral titer reduction from 10^-5.613^ TCID_50_/mL to 10^-2.90^ TCID_50_/mL, which represents a decrease of approximately 10^2.6 ^folds. Results of cytopathic effect and indirect immunofluorescence also indicate a notable decrease in viral infectivity after attapulgite treatment. Additionally, it was observed that modified materials after acidification had weaker antiviral efficacy compared to powdered samples that underwent ultrasonic disintegration, which showed the strongest antiviral effects.

**Conclusion:**

As a result, Attapulgite powders can trap and adsorb viruses to inhibit PEDV in vitro, leading to loss of viral infectivity. This study provides new materials for the development of novel disinfectants and antiviral additives.

**Supplementary Information:**

The online version contains supplementary material available at 10.1186/s12985-024-02396-w.

## Introduction

Porcine Epidemic Diarrhea Virus (PEDV) was initially discovered in the United Kingdom during the 1970s and is responsible for causing Porcine Epidemic Diarrhea (PED). The mortality rate among nursing piglets infected with PEDV is almost 100% [1, 2]. Like COVID-19, PEDV is an RNA virus and a member of the coronavirus family, which is known for its high mutation rate and difficulty in control. In 2013, a PEDV epidemic in the U.S. led to the deaths of 8 million piglets [3–5]. It was demonstrated that traditional inactivated and attenuated vaccines are inadequate in providing immunity against highly lethal PEDV variants, resulting in substantial losses for the global swine industry over the past decade [6, 7]. As the virus continues to mutate and the host range evolves, there is also a potential risk of future human infections [8, 9]. There are currently no commercially available drugs to treat PEDV. Therefore, researchers have been investigating new materials with antiviral properties. The antiviral potential of nanomaterials has attracted considerable attention in recent years. Studies have demonstrated that the sharp edges of graphene can effectively disrupt the structure of PEDV and reduce its infectivity, providing the first evidence of graphene’s antiviral activity [10]. However, there is currently no evidence supporting the antiviral activity of clays such as attapulgite in effectively inhibiting PEDV.

The relative geologic abundance of attapulgite is lower than that of kaolinite and montmorillonite. Nevertheless, attapulgite is characterized by a high density of siloxane groups on its surface, which enables it to undergo reversible adsorption and desorption of water within the zeolite channels through processes such as heating or vacuum exposure. As a fibrous clay, attapulgite possesses strong adsorption capacity and ion exchange properties. Historical records indicate that attapulgite was used as an oral medication for treating diarrhea and pancreatitis during the Mayan period. In modern pharmacology, attapulgite is recognized as an efficacious antidiarrheal and gastrointestinal mucosal protective agent. It has been observed that attapulgite can adhere to the gastrointestinal mucosa, increasing the thickness of the barrier and reducing gastric acid secretion [11–13]. In animal husbandry, attapulgite is used as a feed additive. When consumed by animals, it effectively adsorbs various toxins produced by bacteria in the digestive tract. Studies have demonstrated that attapulgite has a high adsorption rate of 98.2% for *Escherichia coli* and 60% for *Pseudomonas aeruginosa* [14]. The inclusion of 1800 mg/kg of attapulgite in the diet of weaned piglets resulted in a 58.7% reduction in the rate of diarrhea. Attapulgite forms a protective layer on the surface of the intestinal mucosa, preventing bacteria and toxins from directly contacting the intestinal mucosa. This protective action also reduces the abrasion of the intestinal mucosa caused by solid feeds, thereby safeguarding the intestinal villi from damage [14]. Furthermore, research has indicated that attapulgite exhibits strong adsorption of aflatoxin B1 in weakly acidic Sorensen buffer solutions [15].

Attapulgite has great potential in the treatment and prevention of diarrhea diseases. Nevertheless, its antiviral properties have yet to be explored. PEDV is a new outbreak of an old disease that poses an ongoing threat to the global pig industry, so it is essential to study the inhibitory effects of attapulgite on PEDV [16]. Attapulgite has the advantages of low cost and low toxicity. Therefore, the study of the inhibitory effect of attapulgite on PEDV may provide new perspectives for the prevention, control and treatment of PED.

## Methods and materials

### Virus, cells, and materials

Three clay mineral samples were provided by Jiangsu Jinhao New Materials Co., Ltd. These samples include attapulgite ore (designated as SY20210618003), attapulgite powder (designated as SY20210618), and a modified attapulgite variant. The theoretical structural formula for these samples is Mg_5_Si_8_O_20_ (OH) _2_ (OH_2_) _4_ ·4H_2_O. Attapulgite is a safe feed additive ingredient recognized by the EU and China [17–19].

The modified attapulgite sample underwent several processes. It was pre-dried, ultrasonically crushed, acidified with 3% sulfuric acid, and then compressed from the original ore. After standing for over 20 h, it was finally dried and ground to a 200-mesh powder. On the other hand, the attapulgite ore was pre-dried, thoroughly crushed to a particle size smaller than 3 mm, and refined in three stages. The rod-shaped crystals were ultrasonically disintegrated to produce attapulgite powder SY20210618. These rod-shaped crystal bundles underwent pressure filtration, dehydration, strong drying, and powder collection. Both the modified attapulgite sample and the attapulgite powder SY20210618 were sterilized using high-pressure steam before being used for the experiments.

Vero cells from African green monkeys were cultured in Dulbecco’s Modified Eagle Medium (DMEM) containing 10% Fetal Bovine Serum (FBS) and 1% penicillin. IPEC-J2 (intestinal porcine epithelial cells) were cultured in DMEM/F-12(1:1) containing 10% FBS. The PEDV strain (JSX2014, GenBank: MH056658.1) was isolated from a farm in Jiangsu China, multiplied in Vero cells with the genotype GIIb. This virus strain is trypsin incomplete-dependent, with 10 µg/mL trypsin (without EDTA) addition in IPEC-J2 cells but not in Vero cells. The titer of PEDV infection on Vero (without trypsin) was 10^-5.613^ TCID_50_/mL.

### Setting of treatment and control groups

We set up the treatment and control groups using the following methods. In the treatment group, we weighed and autoclaved three kinds of attapulgite and then added a PEDV solution at a concentration of 5%. They were allowed to mix and interact at 4℃ 24 h. The mixture was centrifuged at 5000 ×g for 30 min at 4℃, and the supernatant was filtered through a 0.22 μm filter, which is used as a viral solution for the treatment group and should be stored at -80℃. In the positive control group, the virus solution was untreated PEDV virus solution.

### Detection of viral gene transcription levels

The three attapulgite materials were mixed with PEDV at a concentration of 5% for 24 h to screen for the most effective materials. The viral genome RNA was extracted from both the experimental and control groups. Fluorescent quantitative PCR primers (Table S1) were designed using the registered PEDV *N* gene sequence (NC_003436.1) on GenBank, with β-actin serving as an internal reference. Relative transcription levels of target gene in treatment and control groups were normalized to β-actin transcription and compared using the 2^−ΔΔCt ^method. The One Step TB Green PrimeScript RT-PCR Kit (TAKARA) and *N* gene template were used for one-step fluorescent quantitative PCR to measure PEDV-N mRNA levels.

### Detection of viral protein expression levels

Vero cells were infected with PEDV (MOI = 0.5) collected from the control and experimental groups at a density of 2.4 × 10^5^/mL. After 24 h of virus inoculation, protein lysis buffer was added and shaken for 5 min at room temperature. Then, protein uploading buffer was added and boiled at 95°C for 10 min.

Samples were subjected to SDS-PAGE and transferred to a polyvinylidene fluoride membrane using a wet transfer system. The membrane was blocked with 5% skimmed milk for 2 h at room temperature and incubated overnight at 4°C with a monoclonal antibody against protein S. On the following day, the membrane was incubated with HRP-labeled secondary antibody (anti-mouse). The inhibitory effect of the three attapulgite materials on PEDV infectivity was evaluated using the same method.

Proportionally diluted control virus and experimental group were subjected to fluorescence quantitative PCR assay to ensure the viral titer was the same. Dilutions with the same Ct value as the powder treatment group were selected to infect Vero cells under the same conditions. Western blot was used to detect the difference in PEDV S protein expression levels at the same viral load.

### Concentration and time-dependent experiments

It was set up with four time-gradients and concentration gradients in order to investigate whether attapulgite powder is time and concentration dependent. Vero cells were cultured at a density of 2.4 × 10^5^/mL in 12-well plates. We collected PEDV interacting with attapulgite powder for 1 h, 6 h, 15 h and 24 h at a concentration of 5% and infected Vero cells for 24 h for Western blot, using PEDV N polyclonal antibody (Fig. S1 and S2) as primary antibody. PEDV was mixed with attapulgite powder at concentrations of 0.5%, 2%, 5% and 9% for 24 h and infected Vero cells for 24 h for Western blot, using the same primary antibody.

### Virus titer detection

TCID_50_ was calculated to compare changes in viral titer. The experimental group was attapulgite powder treated virus. PEDV (MOI = 0.5) from the experimental and control groups diluted in a gradient of 10^-1^ to 10^-8^ were inoculated in 96-well plates with a Vero cell concentration of 2.3 × 10^5^/mL. The number of cytopathic effects (CPE) was calculated on the fifth day of infection and TCID_50_ was calculated using the Reed-Muench method.

### Cytopathic effect (CPE)and indirect immune fluorescence (IFA)

In order to fully observe and validate the antiviral effect of attapulgite powder, two types of cells, Vero and IPEC-J2, were used for the CPE and IFA assays. Both of cells were inoculated into a six-well dish at a concentration of 2 × 10^6^/mL. PEDV (MOI = 0.5) was diluted by 10 µg/mL trypsin (without EDTA) and infected with IPEC-J2, while Vero was infected without the addition of trypsin. CPE was observed 24 h after Vero infection and 48 h after IPEC-J2 infection.

After 12 h of infection, the maintenance solution was discarded. The cells were then rinsed three times with PBS and then fixed for 10 min by adding 4% paraformaldehyde. The cells were permeabilized with 0.5% Triton X-100 and blocked with 5% BSA at room temperature, and a polyclonal antibody against PEDV N protein (Fig. S1 and S2) prepared in our laboratory was used as primary antibody. FITC-labeled fluorescent secondary antibody was added in the dark and observed under a fluorescent inverted microscope.

#### Transmission electron microscopy (TEM)

In order to compare the differences in the transmission electron microscopy images of the virus before and after treatment with Attapulgite, the PEDV virus solution was treated by the following two methods. Experimental group: PEDV solution was purified by sucrose density gradient centrifugation and treated with 5% concentration of attapulgite powder. Control group: PEDV virus solution was purified by sucrose density gradient centrifugation without material treatment. After two rounds of crude extraction, PEDV was purified in three sucrose gradient solutions of 60%, 40% and 20%.

Copper mesh was glued to filter paper, vortexed to mix the purified viral solution, and then dropped onto a carbonized copper mesh, adsorbed and left to stand for a few minutes for 1∼2 min of negative staining. Images of virus particles before and after treatment were captured using a Hitachi HT7700 transmission electron microscope at 1:500 nm and 1:200 nm.

### Statistical analysis

Quantification data represent the means and standard deviations from multiple independent experiments, as indicated for the individual assays. Statistical analysis was performed using GraphPad Prism version 8.1.0, with differences between groups analysed using one-way ANOVA. Significance cut-offs were defined as: ns: no significance, 0.05<*P* ≤ 0.10: moderate significance, 0.01<*P* ≤ 0.05: strong significance, *P* ≤ 0.01: very strong significance.

## Results and discussion

### Characterization of attapulgite materials

Attapulgite is composed of continuous two-dimensional tetrahedral layers and discrete octahedral layers, which form parallel structural channels along the clay fibers. It has a density of 2.1∼2.3 g/cm^3^, a molecular weight of 583.38 g/mol, and a specific surface area of 130 m^2^/g [20–23]. The combination of its high aspect ratio, abundant surface silanol groups, and natural structural pores gives it outstanding physical properties, such as a large surface area, strong ion exchange capacity, and numerous surface adsorption sites [24].

**Fig. 1:**
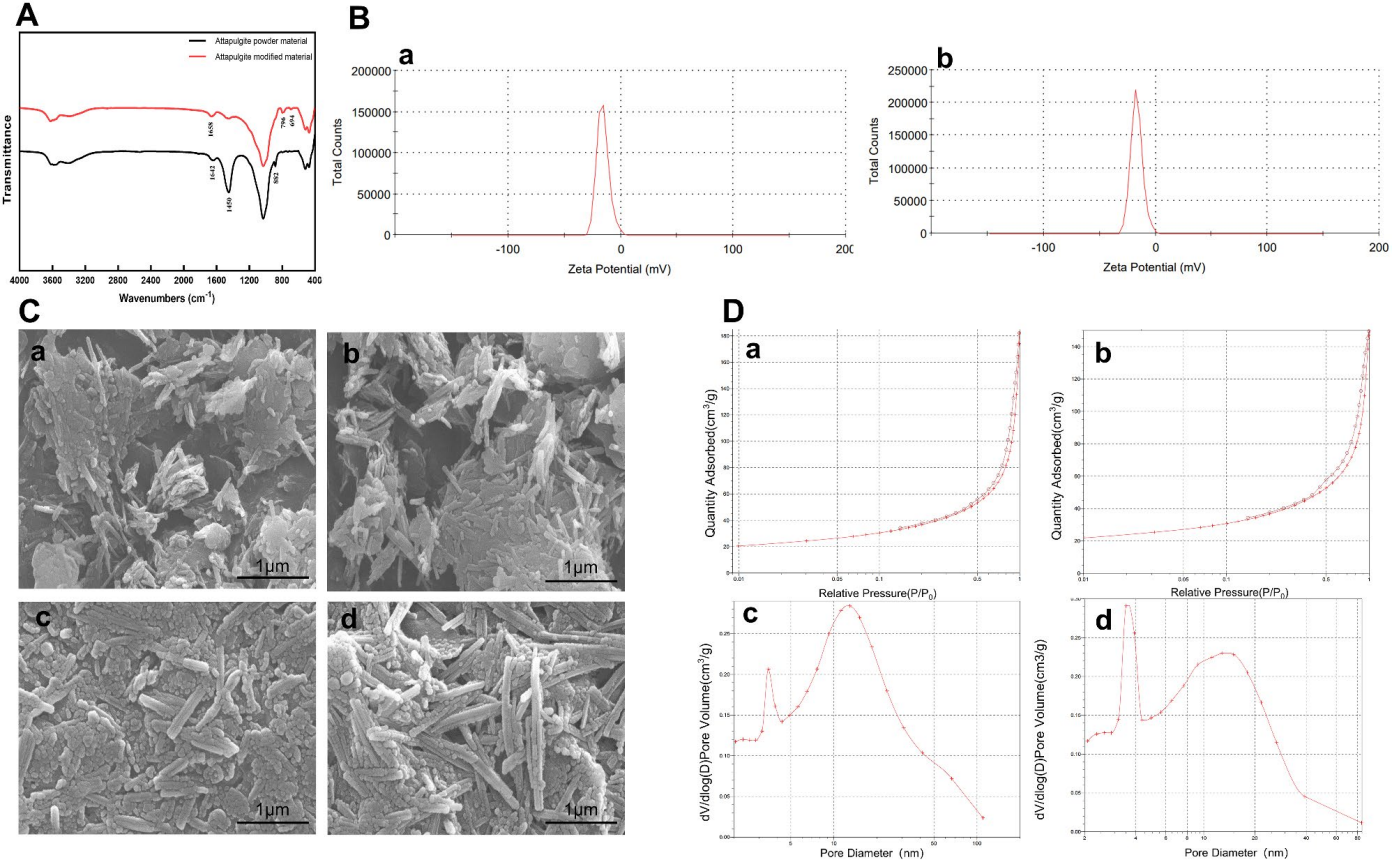
Characteristics of materials. (**A**) FTIR spectra of attapulgite powder and modified attapulgite. (**B**) ZETA potential of attapulgite powder (**a**) and modified attapulgite (**b**). (**C**) SEM images of attapulgite powder (**a**), modified attapulgite (**b**), PEDV with attapulgite powder (2000×) (**c**), and PEDV with modified attapulgite (2000×) (**d**). (**D**) N_2_ adsorption-desorption isotherms of attapulgite powder (**a**) and modified attapulgite (**b**). Pore size distribution curves of attapulgite powder (**c**) and modified attapulgite (**d**)

The X-ray fluorescence measurements of the attapulgite materials used in this study are presented in Table 1. Both materials consist mostly of SiO_2_, accounting for approximately 57% in modified attapulgite and 46% in powdered attapulgite. The SiOH groups on the surface of SiO_2_ enable strong adsorption and hydrogen bonding with oxygen and nitrogen atoms. Both materials contain about 13% Al_2_O_3_. The MgO content in powdered material is 9%, while in modified material, it is 5%. MgO is a hygroscopic alkaline oxide that readily reacts with other substances to form a strong adsorbent called Mg(OH)_2_. These elemental characteristics, combined with the porous structure of attapulgite, suggest that these materials have strong adsorption capabilities and an affinity for PEDV. The infrared spectroscopy analysis of powdered and modified attapulgite is shown in Fig. 1. In the powdered attapulgite sample, the stretching vibration band of Al-OH-M is located at 882 cm^-1^, and the characteristic absorption peak of CO_3_^2-^ appears at 1450 cm^-1^. In the modified attapulgite sample, the infrared spectrum shows that the stretching vibration band of Al-OH-M is located at 694 and 796 cm^-1^, while the stretching vibration bands of free hydroxyl groups in both materials are at 3650 –3600 cm^-1^ (Fig. 1A). Both powdered and modified attapulgite exhibit negative Zeta potentials (Fig. 1B). Both materials follow type IV isotherms with H3-type hysteresis loops (Fig. 1D). Pore size distribution curves reveal that the modified material has slightly larger pores compared to the powdered material. Further investigation of the morphological differences between powdered and modified attapulgite was conducted using scanning electron microscopy, as shown in Fig. 1C. The powdered attapulgite exhibits typical rod-like crystallites and bundles. The surfaces of the rod-shaped crystals are smooth, with no protrusions, indicating a lower degree of dissociation compared to the powdered material. Both the number and size of aggregates are larger in the modified material compared to the powdered material. Figure 1C (c and d) presents Scanning electron microscope (SEM) images of attapulgite treated with PEDV. The powdered rod-shaped crystals adsorb a substantial amount of protein, impurities, and viruses, demonstrating the material’s stronger adsorption capacity compared to the modified material.

**Table 1:** Chemical composition of Attapulgite powder and modified attapulgite

Sample name	Attapulgite powder	Modified attapulgite
MgO	9.05%	5.59%
Al_2_O_3_	12.80%	13.22%
SiO_2_	46.68%	57.66%
CaO	9.57%	2.32%
Fe_2_O_3_	8.46%	7.22%

### PEDV *N* Gene transcription levels and s protein expression levels

**Fig. 2:**
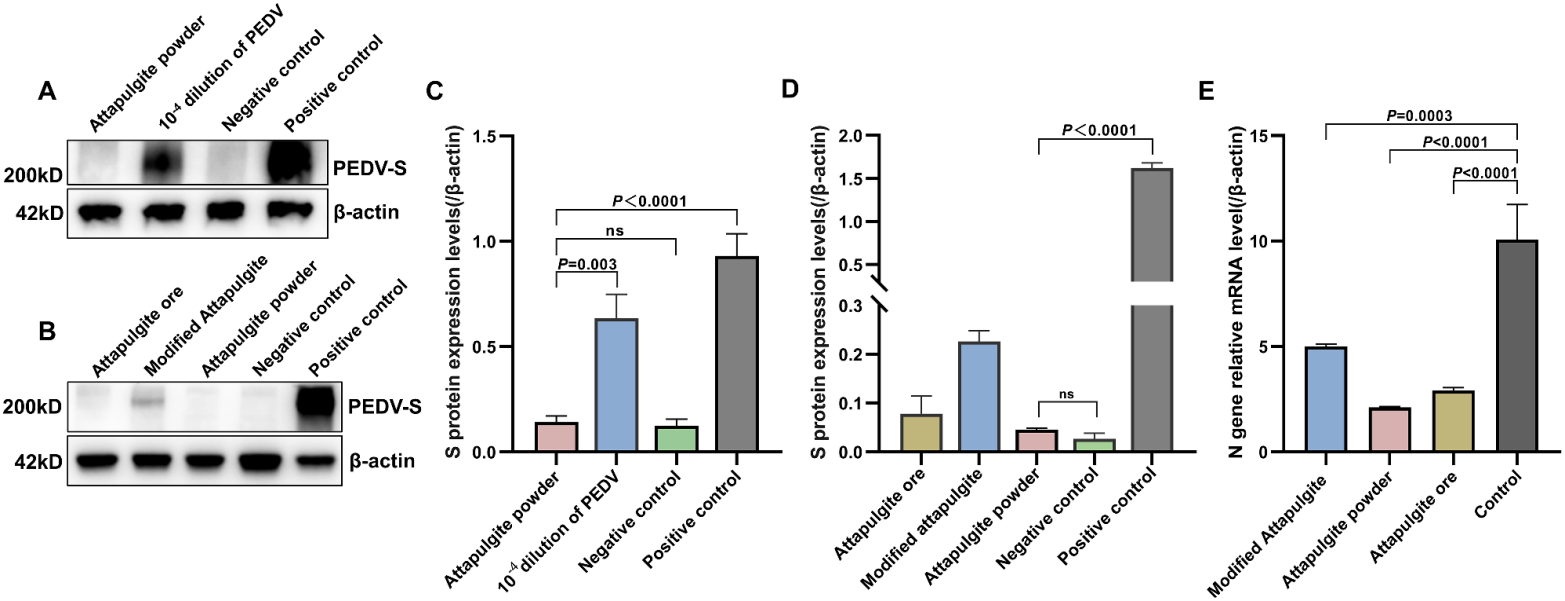
Screening results of three clay materials (interact with PEDV at 5% concentration for 24 h). Western blot and Grayscale analysis of 10^-4^ dilution group (**A** and **C**) and three materials treatment group (**B** and **D**). The three materials treatment and control groups of relative mRNA level of *N* gene (**E**). (ns: no significance, *P* ≤ 0.01: very strong significance)

The target gene for detection was selected as the *N* gene encoding the PEDV nucleocapsid protein. The *N* gene is highly conserved and plays a role in viral replication by providing a nuclear localization signal. Additionally, the N protein is used for early diagnosis of PEDV infection [25, 26]. The differential transcription levels of the PEDV *N* gene was calculated using 2^−ΔΔCt^ method, as shown in Fig. 2E and Table S2. The powder and ore groups showed highly significant differences from the control group, and the difference between the modified and control groups was significant. The results showed that all three types of attapulgite clay materials inhibited PEDV, with the strongest inhibition in the powdered material and the weakest inhibition in the modified material. Such differences may be related to the fact that the modified materials were treated with sulfuric acid. From the level of *N* gene transcription, powder showed better inhibition than modification, which was attributed to the purification method of the original ore.

The S protein is encoded by the PEDV *S* gene and consists of the S1 and S2 subunits. It identifies and binds to receptors during the virus infection process, facilitating membrane fusion. In this study, the expression of the S protein after treatment with powdered attapulgite was found to be consistent with the negative control level, indicating a significant reduction or loss of viral infectivity (Fig. 2B and D). However, the virus treated with modified attapulgite still showed some infectivity. To further validate the inhibitory effect of powdered attapulgite on viral infectivity, the viral solution from the control group was diluted and subjected to RT-qPCR. A diluent with the same Ct value as the powdered sample was selected. Vero cells were infected with the diluted control and the sample treated with an equal concentration of powdered attapulgite, and the expression of the S protein was detected using Western blot. As shown in Fig. 2A and C, under the same virus concentration, a significant difference was found between the dilution and treatment groups, suggesting that the inhibition of S protein results in reduced viral infectivity. The S protein plays a critical role in the virus’s ability to enter host cells and is a major immunogenic protein of PEDV. Attapulgite can inhibit or disrupt the expression of the S protein, weakening its infectivity during the early stages of PEDV infection and effectively inhibiting PEDV infection.

### Time and concentration dependency

**Fig. 3:**
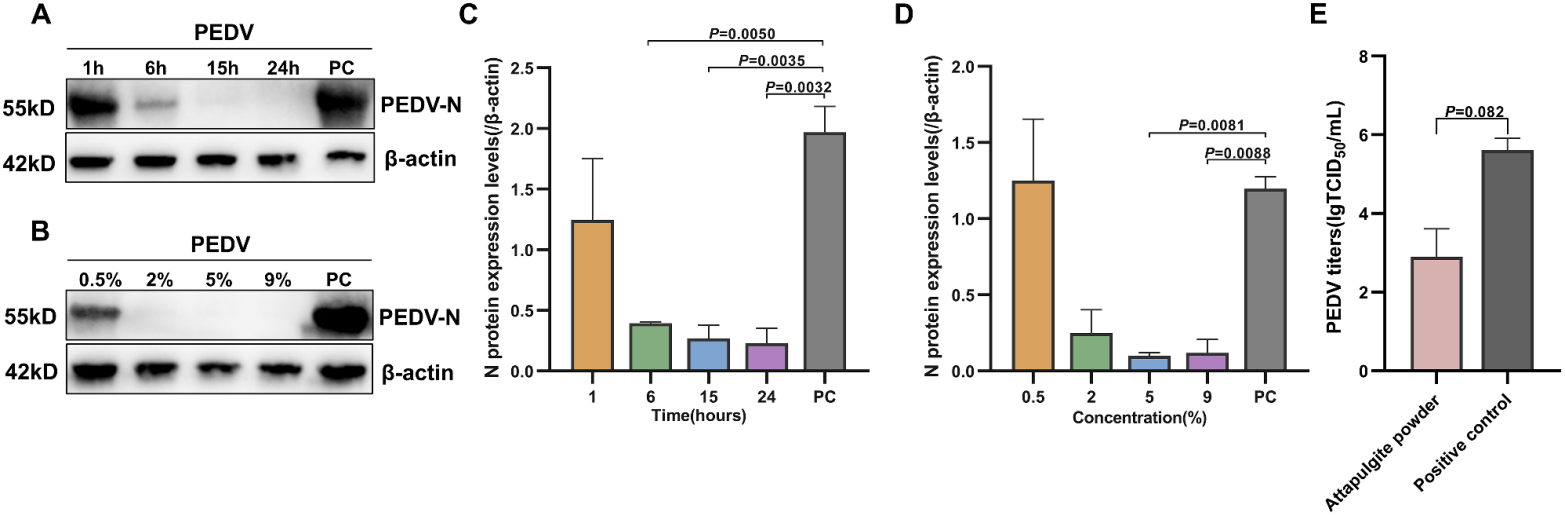
Results of time and concentration dependence of attapulgite powder-treated PEDV and change of PEDV titers. Western blot results and grayscale analysis of PEDV-infected Vero cells treated with different times (**A** and **C**) and different concentrations (**B** and **D**) of powdered attapulgite. Histogram of PEDV viral titers in control and treatment groups (**E**). (PC: positive control. ns: no significance, 0.01<*P* ≤ 0.05: strong significance, *P*<0.01: very strong significance)

We investigated the inhibitory effect of attapulgite powder on PEDV at different concentrations and for different times. PEDV interacted with 5% attapulgite powder for 1, 6, 15 and 24 h to infect Vero cells. We detected N protein expression levels by Western blot (Fig. 3A). The 6, 15 and 24 h groups showed highly significant differences from the control group, which showed the smallest *P*-value in the 24 h group (Fig. 3C). Similarly, PEDV interacted with 0.5%, 2%, 5% and 9% attapulgite powder for 24 h to infect Vero cells. The results showed that PEDV was effectively inhibited at both 5% and 9% concentrations, when combined with gray scale analysis, the *P*-value of 5% was the smallest (Fig. 3B and D). Based on the time and concentration gradient experiments, we concluded that 5% attapulgite powder interacting with PEDV for 24 h was the most suitable experimental condition.

### PEDV titers

To determine the viral titers of the powdered samples after treatment, we measured the TCID_50_ of each viral solution. After 5 days of cultivation, we recorded the number of lesions and non-lesions. We calculated the lesion rate, and the results are presented in Fig. 3E. According to the Reed-Muench method, the average viral titer of the control group was determined to be 1 × 10^-5.613^ TCID_50_/mL, while that of the powdered group was 1 × 10^-2.90^ TCID_50_/mL, indicating a 10^2.6^ folds difference (Table S3 and S4). This indicates that the virulence of PEDV is significantly reduced or lost after treatment with powdered attapulgite compared to untreated virus. This finding is consistent with the Western blot results, and showed that attapulgite disrupts PEDV S protein, resulting in reduced viral infectivity and titers.

### CPE and IFA

**Fig. 4:**
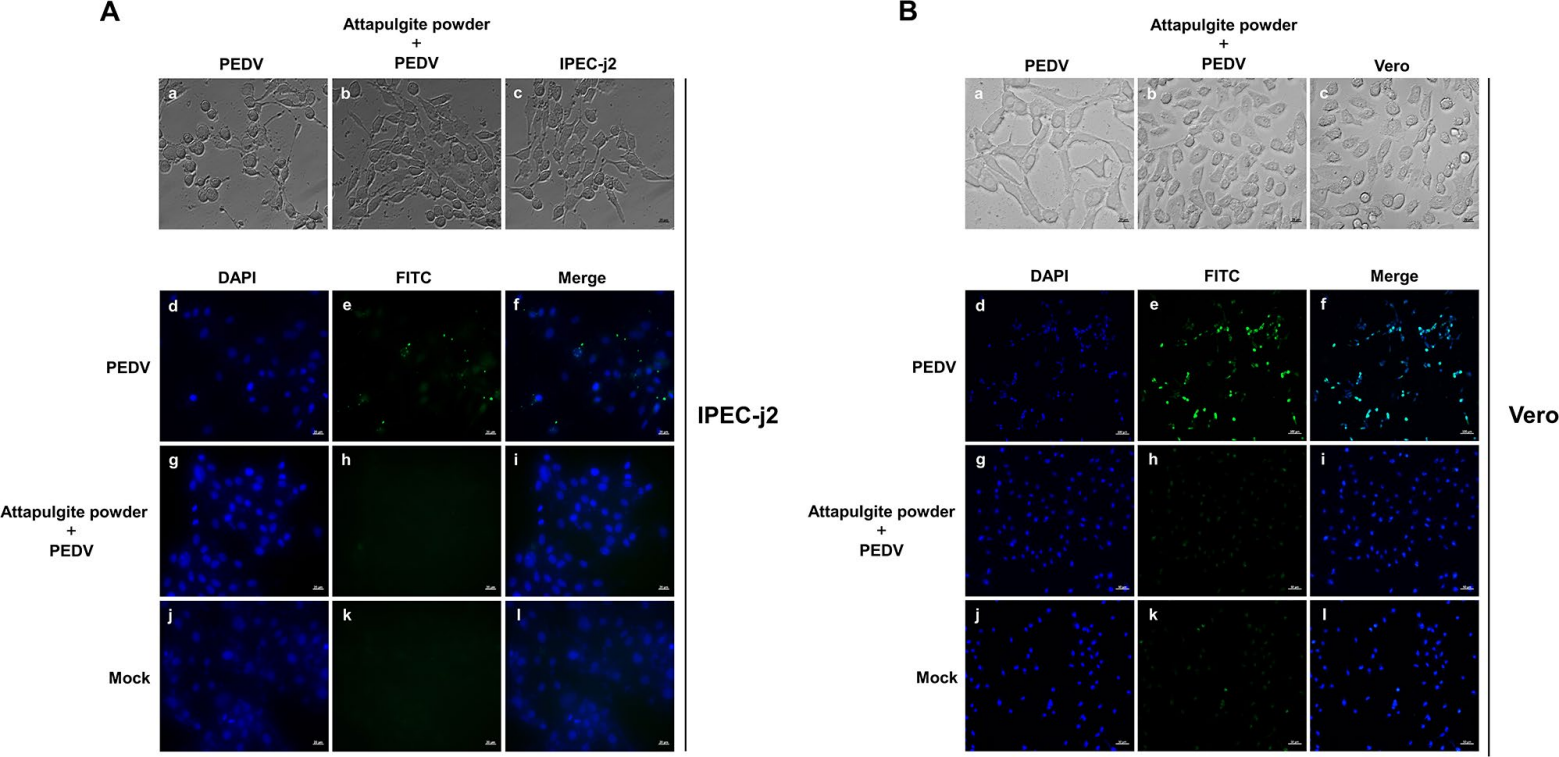
CPE and IFA of Vero and IPEC-J2. CPE and IFA assay using IPEC-J2 (**A**) and Vero (**B**) cells infected with PEDV in the presence and absence of powdered attapulgite. Extent of lesions and fluorescence intensity on two types of cells: cells infected with PEDV (a, d, e and f); PEDV-infected cells in the presence of powdered attapulgite (b, g, h and i); Mock-infected cells (c, j, k and l). Vero and IPEC-J2 (add 10 µg/mL trypsin (without EDTA)) were infected with PEDV at MOI = 0.5

Vero and IPEC-J2 cells were infected with PEDV from the treatment and control groups, respectively, and subsequently performed CPE and IFA. Notably, we added trypsin (without EDTA) when PEDV was infected with IPEC-J2. We observed under the microscope that Vero cells showed indentation, crumpling and lysis, which were infected by the positive group for 24 h (Fig. 4Ba). Meanwhile IPEC-J2 was infected by the positive group for 48 h, the cell morphology became rounded, crumpled and lysed (Fig. 4Aa). In contrast, no significant CPE was seen in both types of cells in the negative and treated groups, which visually demonstrates the inhibitory effect of powdered attapulgite (b and c of Fig. 4A and B).

To observe the fluorescence intensity of PEDV particles after treatment with powdered attapulgite, we used a Leica DM500 inverted fluorescence microscope. In this part, we did not use PEDV S protein monoclonal antibodies as primary antibodies due to their single binding site, which resulted in suboptimal experimental results. The treatment and control PEDV groups were inoculated with Vero and IPEC-J2 under the same conditions as in the CPE assay. Green fluorescence signals were observed in both control groups (d, e and f of Fig. 4A and B), while the treatment groups (g, h, i of Fig. 4A and B) fluoresced very weakly close to the negative group (j, k and l of Fig. 4A and B).

### TEM analysis

**Fig. 5:**
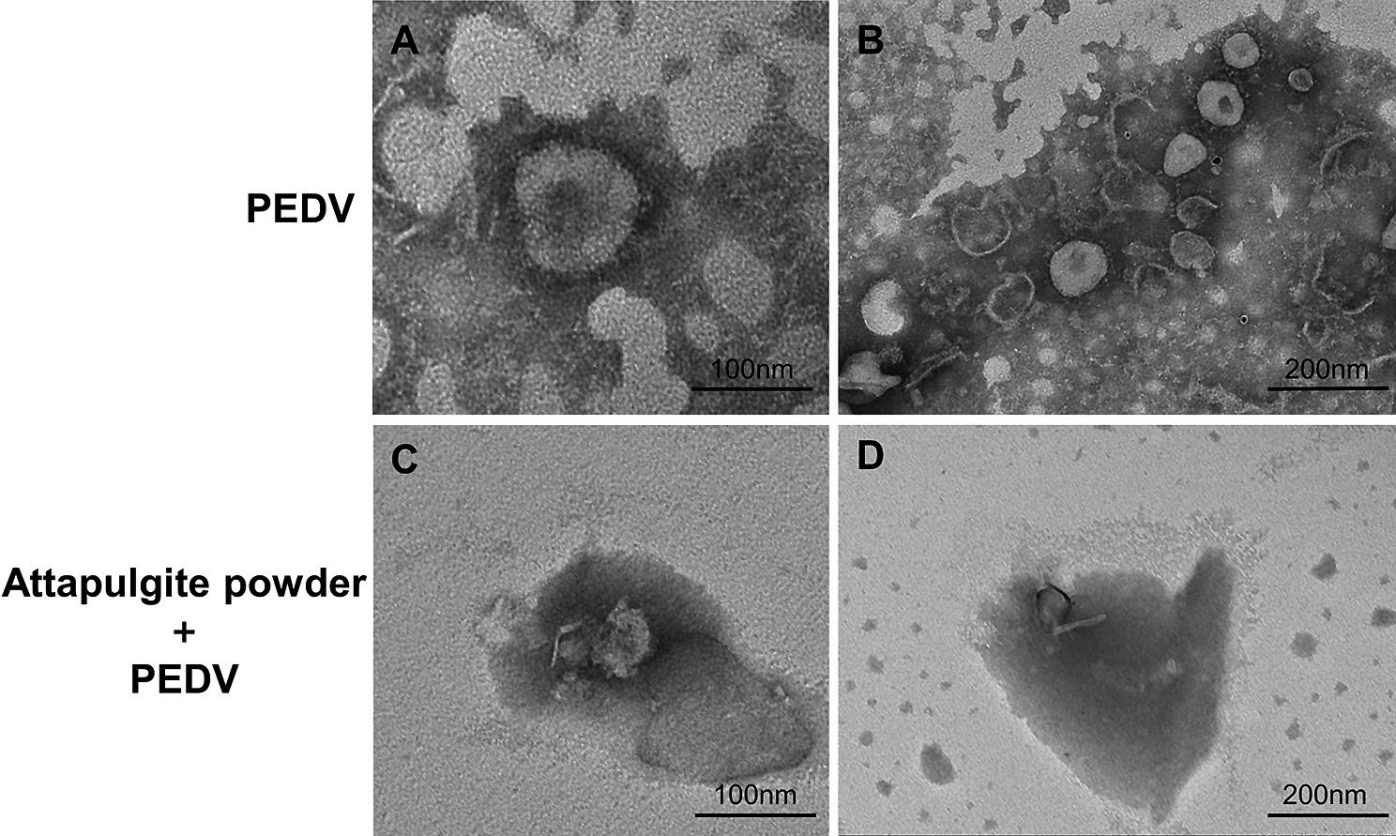
TEM of PEDV- attapulgite powder interactions. TEM images of PEDV (**A** and **B**) and PEDV treated by attapulgite powder (**C** and **D**). Both groups of viruses were purified by sucrose density gradient centrifugation

The morphology of PEDV particles was observed using a Hitachi HT7700 transmission electron microscope. The particles exhibited the typical coronavirus morphology, with most of them being spherical or ellipsoidal in shape. The envelope of the particles had radially arranged fibrous projections, measuring 18∼23 nm in length. The average diameter of the viral particles was approximately 130 nm [27]. Figure 5A and B showed transmission electron microscope images of the control group before treatment with attapulgite powder, revealing the intact structure of PEDV particles. These particles are round or elliptical with crown-like spike outside. The viral particles appeared to be highly intact, with multiple PEDV particles (usually between 2 and 6) observed in the same field of view [27, 28]. Figure 5C and D showed a brighter background compared to the pre-treatment images, indicating a significant reduction in impurities and protein content. At the same time, we could observe both the defective PEDV particles and the intact attapulgite powder rod crystal structures, which suggests that augite powder was able to adsorb viral particles and disrupt their structures, similar to the inhibitory effect of the carbon-based nanomaterial graphene on PEDV.

## Discussion

PED, as a representative of the new outbreak of an old disease, brings economic losses to the farming industry. With the changing host spectrum of the virus, there is even a risk of infecting humans in the future. Therefore, the development of materials to inhibit PEDV is necessary to prevent PED outbreaks. On the other hand, Attapulgite is an inexpensive, naturally occurring hydrated magnesium-aluminosilicate clay mineral formed by weathering and geological action of surface silicate minerals [29]. Due to its large specific surface area and micro- or mesoporous structure, magnesia-aluminosilicate clay is widely used as an adsorbent for a variety of applications [30–32].Chen et al. demonstrated that Attapulgite can be used as a potential hemostatic agent with hygroscopic effect, concentration of clotting factors, and formation of porous composite sponges for rapid hemostasis when combined with chitosan [33].

Attapulgite exhibits excellent antimicrobial effect as a fiber clay. Pristine sepiolite and palygorskite possess limited antibacterial activities although varying results were obtained on fibrous clays of different origin and purity [34, 35]. Fibrous clays with high specific surface area can act as efficient adsorbents for pathogens and may cause physical damage to membrane [36–38]. In addition, the cavity structure (channels and tunnels) of clay is capable of immobilizing certain natural antimicrobial components such as quaternary ammonium salts, ethylene oxide, and nano-silver ions to produce a sustained and stronger antimicrobial effect [39]. Attapulgite was listed in the feed additives catalog in the Official Journal of the European Union and the Bulletin of the Ministry of Agriculture of the People’s Republic of China, which proves that Attapulgite does not have cytotoxicity and its safety is beyond doubt [17, 18]. In this study, Attapulgite was insoluble in all cell culture related solutions so we did not perform cytotoxicity tests, but its safety as a feed additive can be assured [19].

The efficient antimicrobial properties of clay have been widely explored, however, there are no reports on its antiviral properties. Therefore, we used PEDV as a model pathogen and demonstrated for the first time that Attapulgite could inhibit PEDV efficiently at the gene transcription level and expression level. Meanwhile, we also found that there were differences in the effects of the three materials on PEDV, and evaluated them in terms of viral titer, infectious ability, and viral copies, and screened out Attapulgite, the powder with the best effect in inhibiting PEDV. Ye et al. first explored the antiviral properties of Graphene oxide (GO) and (reduced graphene oxide) rGO (in the case of PEDV and PRV), which showed similar antiviral activity, suggesting that the antiviral mechanism of GO may be attributed to the negative charge and nanosheet structure [40].

**Fig. 6:**
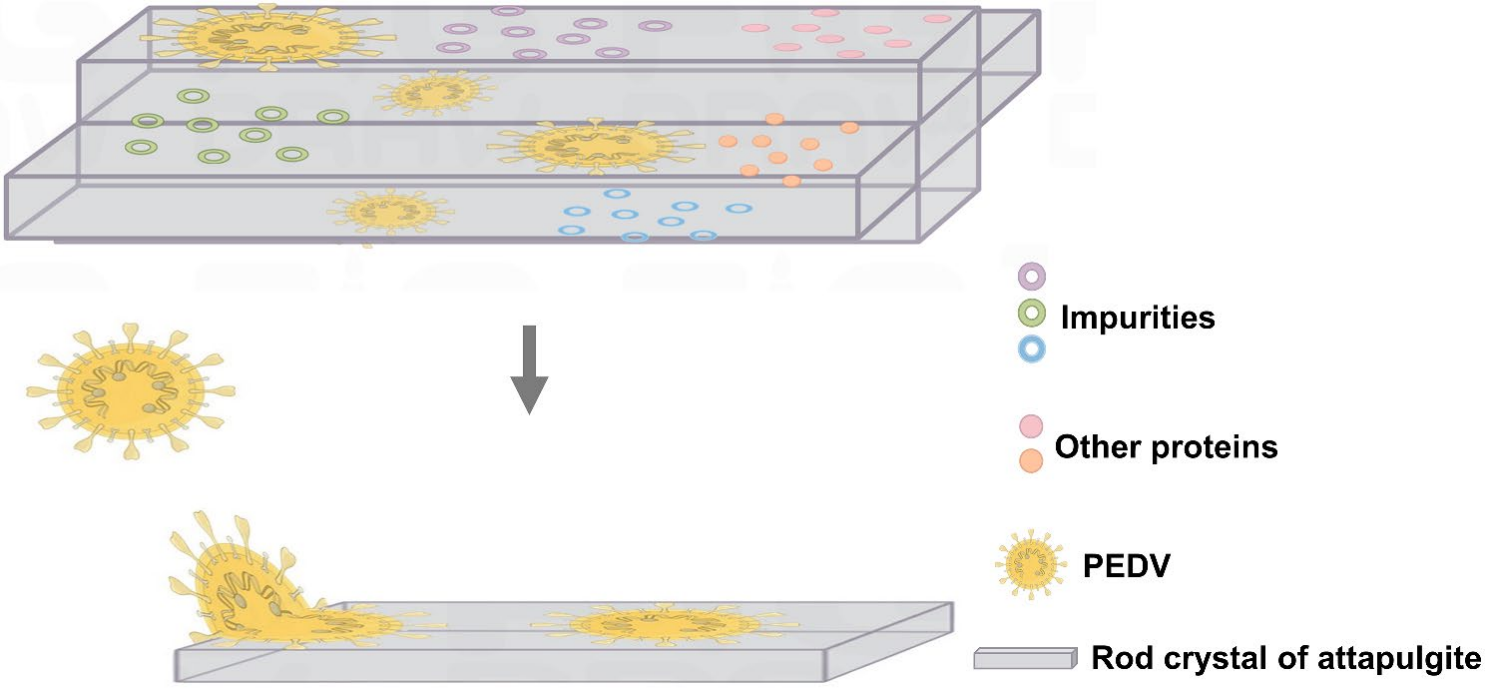
Simple schematic of Attapulgite adsorption of PEDV. Attapulgite has strong adsorption properties to adsorb PEDV to the surface of the rod crystal including impurities and other proteins

Under TEM, virus particles are adsorbed and destroyed, which is one of the important mechanisms by which the powder materials inhibit PEDV. Comparing the SEM images of the powder attapulgite and the modified attapulgite, the findings indicate that the rough surface of the powder facilitates the capture and adhesion of PEDV virus particles. We drew a simple schematic diagram of PEDV adsorption by attapulgite, in which the rod-crystal structure of attapulgite makes up the pore, but PEDV is mainly adsorbed on the surface of it as well as some impurities and proteins (Fig. 6). The Al-(OH) of the powders have the effect of facilitating virus contact, while the large negative charges carried on the surface also play an important role in the antiviral properties. This may be contrary to the conclusion that nanomaterials are antimicrobial, and it has been demonstrated that the positive charge on the surface of certain composite nanomaterials can enhance the antimicrobial activity, e.g., graphene-silver nanoparticle composites and Qacos/ZnO/PAL have elaborated on the antimicrobial effect of the positive charge carried by the materials [38, 41–43](H. As for the antiviral aspect, it has been shown that graphene has good antiviral properties when negatively charged, but graphene-silver nanoparticle composites with positive charges do not exhibit antiviral activity [40].

## Conclusions

This study demonstrated the antiviral properties of attapulgite clay, specifically that powdered attapulgite had the strongest inhibitory effect. We found that attapulgite-treated PEDV became less infectious in Vero and IPEC-J2 cells by viral titer, CPE and IFA assays. It was observed that the antiviral activity of attapulgite nanomaterials was weakened by acidification, whereas powered attapulgite prepared via ultrasonic dissociation showed the strongest antiviral effect. It was observed by TEM analysis that attapulgite disrupted the structure of virus particles, so we inferred that attapulgite reduces infectivity by adsorbing and disrupting viruses. Notably, both PEDV and attapulgite clay surfaces were negatively charged, suggesting that the adsorption capacity may be due to hydrophobic or VDE (van der Waals’ forces) rather than electrostatic forces. In conclusion, this study validated the antiviral properties of attapulgite clay, revealed the differences in antiviral activity due to different modification methods, and inferred the potential mechanism of the antiviral effects of attapulgite clay. These findings broaden the scope of application of attapulgite clay and provide valuable insights for future research and development of environmental antiviral agents.

### Electronic supplementary material

Below is the link to the electronic supplementary material.

Supplementary Material 1

## Data Availability

No datasets were generated or analysed during the current study.

## Abbreviations

CPE: Cytopathic Effect
IFA: Indirect immune fluorescence
PEDV: Porcine epidemic diarrhea virus
PED: Porcine epidemic diarrhea
PAL: Palygorskite
Qacos: Quaternary ammonium chitooligosaccharide
SEM: Scanning electron microscope
TEM: Transmission electron microscope
VDE: Van der Waals’ forces
