# Supplementary material for: Virological evaluation of natural and modified attapulgite against porcine epidemic diarrhoea virus
Source: Virol J. 2024 May 30;21:120. doi: 10.1186/s12985-024-02396-w (PMC11137985; doi:10.1186/s12985-024-02396-w)
Supplement: Supplementary file 1 — Supplementary Material 1 [file 12985_2024_2396_MOESM1_ESM.docx]

**Table S1:** Primer sequences for RT-qPCR

| Primer name | Primer sequence |
| --- | --- |
| PEDV-N-F | 5’-ATGGCTTCTGTCAGCTTTCAGG -3' |
| PEDV-N-R | 5’-ATTTCCTGTATCGAAGATCTCGTTG -3' |

**Table S2:** Results of RT-qPCR

| Group | Ct values | | |
| --- | --- | --- | --- |
| Treatment 1 | 20.07 | 20.55 | 20.70 |
| Treatment 2 | 29.98 | 29.81 | 29.77 |
| Treatment 3 | 26.51 | 26.94 | 27.45 |
| Control | 9.45 | 9.36 | 9.87 |

**Table S3:** Results of TCID50 measurement in the control group

| Dilutability | 1 | | | 2 | | | 3 | | |
| --- | --- | --- | --- | --- | --- | --- | --- | --- | --- |
|  | CPE | CPE free | CPE% | CPE | CPE free | CPE% | CPE | CPE free | CPE% |
| 10^-1^ | 8 | 0 | 100% | 8 | 6 | 100% | 8 | 0 | 100% |
| 10^-2^ | 8 | 0 | 100% | 8 | 7 | 100% | 8 | 0 | 100% |
| 10^-3^ | 8 | 0 | 100% | 8 | 6 | 100% | 8 | 0 | 100% |
| 10^-4^ | 4 | 4 | 64% | 3 | 8 | 69% | 8 | 0 | 100% |
| 10^-5^ | 0 | 8 | 20% | 0 | 4 | 38% | 2 | 6 | 45% |
| 10^-6^ | 1 | 7 | 14% | 0 | 3 | 27.6% | 2 | 6 | 20% |
| 10^-7^ | 1 | 7 | 7% | 1 | 6 | 22.2% | 1 | 7 | 5% |
| 10^-8^ | 0 | 8 | 3% | 2 | 7 | 17% | 0 | 8 | 0% |
|  | 10^-5.318^ TCID_50_/mL | | | 10^-5.612^ TCID_50_/mL | | | 10^-^^5.909^ TCID_50_/mL | | |

**Table S4:** Results of TCID50 measurement in the treatment 2

| Dilutability | 1 | | | 2 | | | 3 | | |
| --- | --- | --- | --- | --- | --- | --- | --- | --- | --- |
|  | CPE | CPE free | CPE% | CPE | CPE free | CPE% | CPE | CPE free | CPE% |
| 10^-1^ | 0 | 8 | 33.3% | 2 | 6 | 74% | 1 | 7 | 59% |
| 10^-2^ | 1 | 7 | 21% | 1 | 7 | 54% | 0 | 8 | 37.5% |
| 10^-3^ | 1 | 7 | 12% | 2 | 6 | 42% | 1 | 7 | 28% |
| 10^-4^ | 0 | 8 | 6.25% | 0 | 8 | 31% | 1 | 7 | 21% |
| 10^-5^ | 0 | 8 | 5% | 4 | 4 | 28% | 1 | 7 | 16% |
| 10^-6^ | 1 | 7 | 4.2% | 5 | 3 | 19% | 1 | 7 | 12% |
| 10^-7^ | 1 | 7 | 1.89% | 2 | 6 | 6.9% | 3 | 5 | 9% |
| 10^-8^ | 0 | 8 | - | 1 | 7 | 2.1% | 2 | 6 | 4% |
|  | - | | | 10^-3.4^ TCID_50_/mL | | | 10^-2.4^ TCID_50_/mL | | |


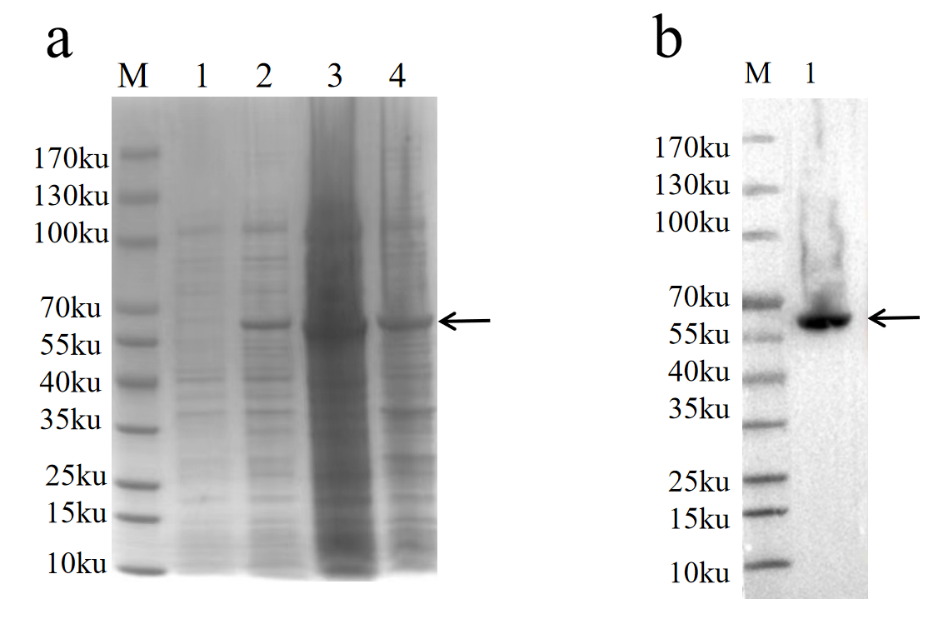


**Fig. S1: Identification of PEDV N protein expression form and WB identification of recombinant protein PEDV-N after purification**

a: Identification of the expression form of PEDV N recombinant protein. M: Protein molecular quality standards; 1: Before PEDV-N induction; 2: After induction of PEDV-N; 3: PEDV-N protein soluble expression product; 4: PEDV-N insoluble expression product

b: Western-blot identification of PEDV-N. M: Protein molecular quality standards; 1: Purified PEDV-N protein


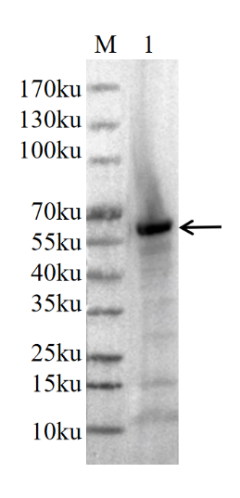


**Fig. S2: Identification of binding ability of mouse polyclonal antisera to PEDV N protein**

M: Protein molecular quality standards; 1: N-protein multi-antiserum binding to PEDV


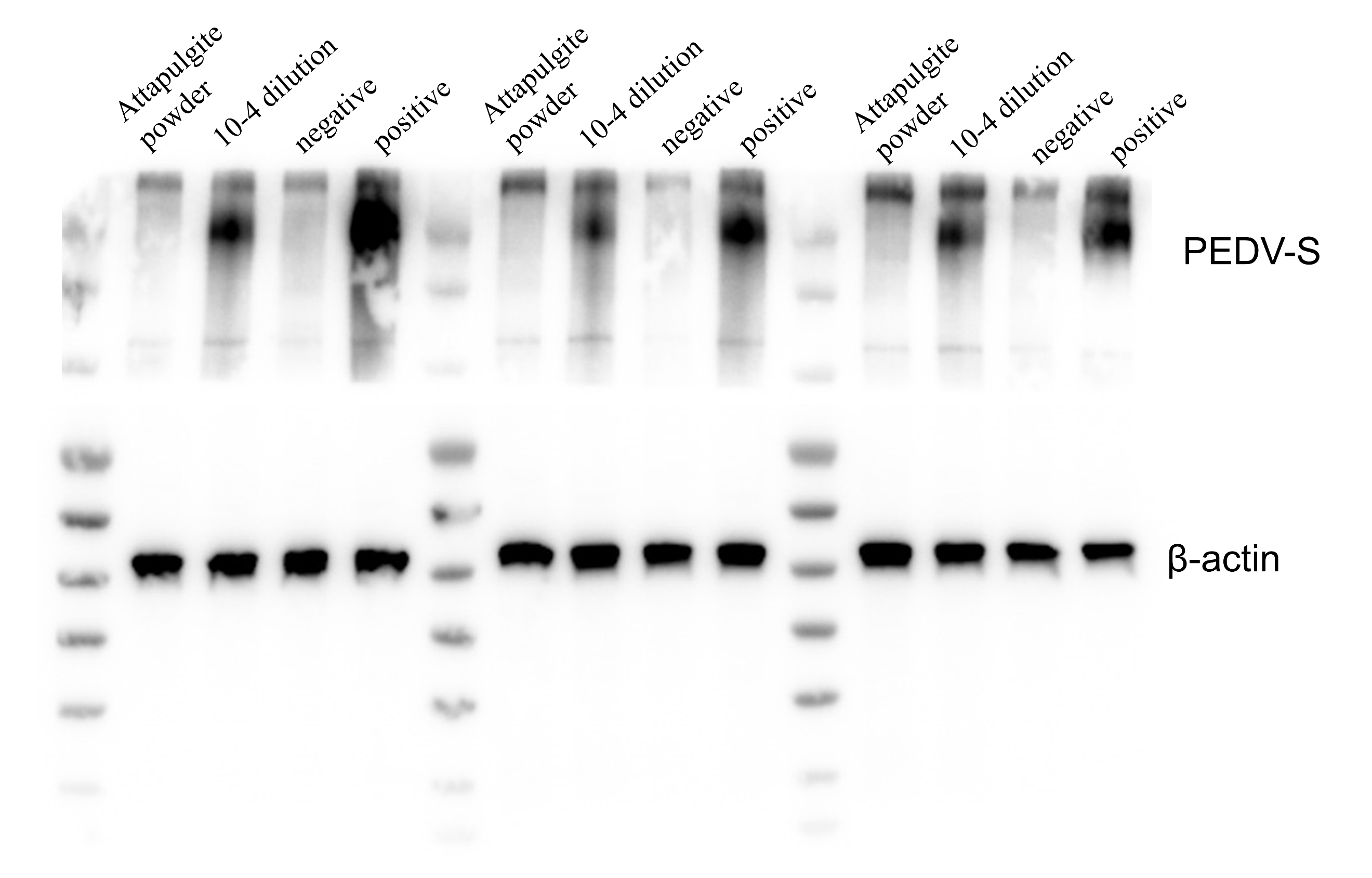


**Fig. S3: Uncropped blots image of figure 2A**


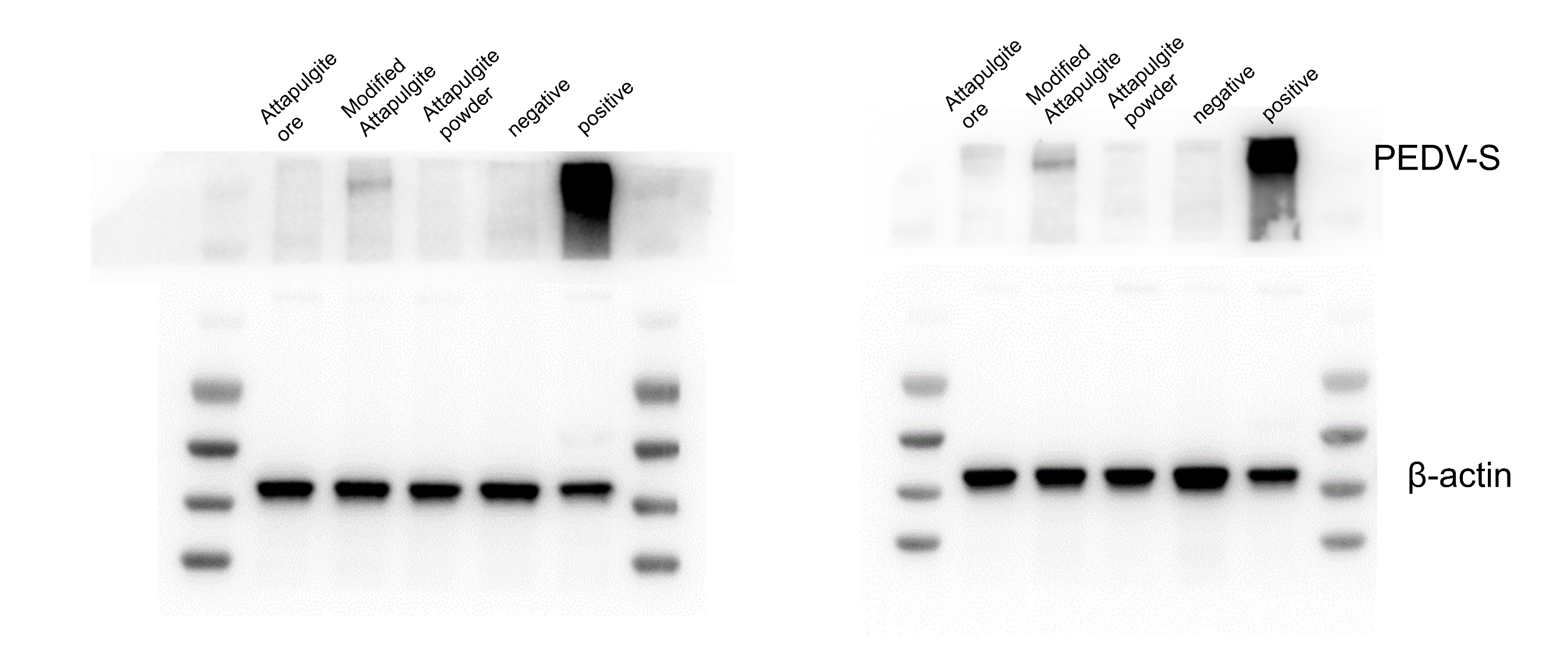


**Fig. S4: Uncropped blots image of figure 2B**

**
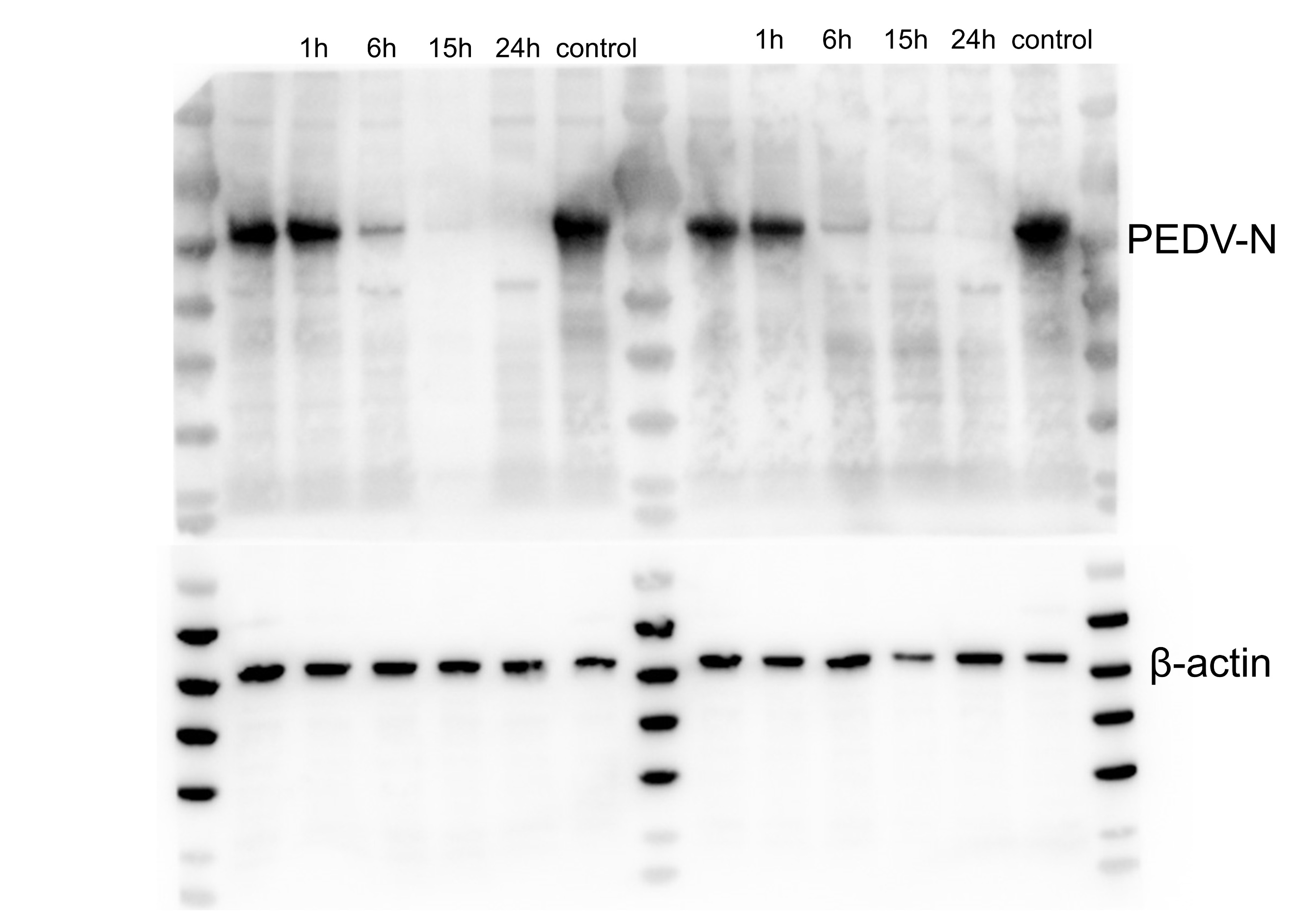
**

**Fig. S5: Uncropped blots image of figure 3A**


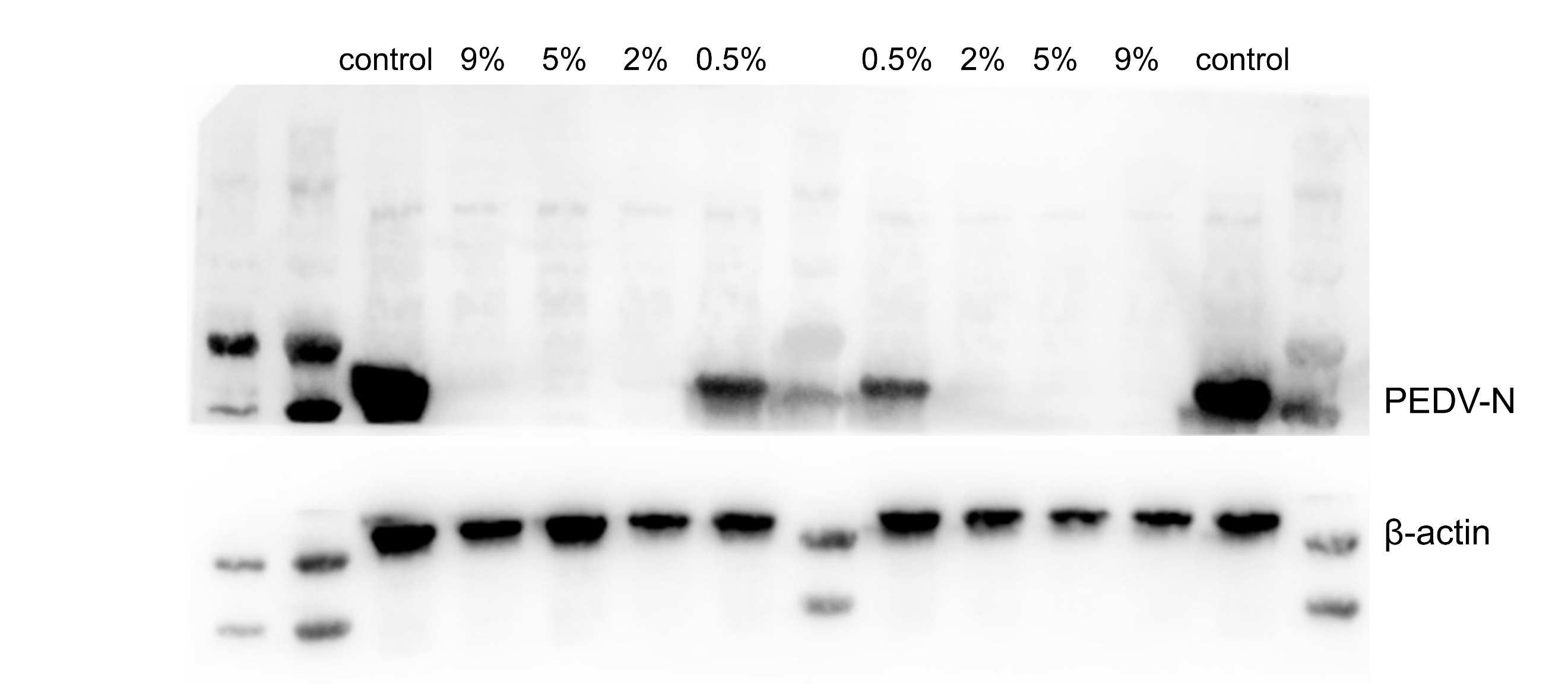


**Fig. S6: Uncropped blots image of figure 3B**
